# Modular air–liquid interface aerosol exposure system (MALIES) to study toxicity of nanoparticle aerosols in 3D-cultured A549 cells in vitro

**DOI:** 10.1007/s00204-023-03673-3

**Published:** 2024-02-10

**Authors:** M. J. Küstner, D. Eckstein, D. Brauer, P. Mai, J. Hampl, F. Weise, B. Schuhmann, G. Hause, F. Glahn, H. Foth, A. Schober

**Affiliations:** 1 Department of Nano-Biosystems Engineering, Institute of Chemistry and Biotechnology, Ilmenau University of Technology, P.O. Box, 98684 Ilmenau, Germany; 2 Institute of Environmental Toxicology, Martin-Luther-University Halle-Wittenberg, 06108 Halle (Saale), Germany; 3 Biocenter, Department of Electron Microscopy, Martin-Luther-University Halle-Wittenberg, 06099 Halle (Saale), Germany

**Keywords:** Lung aerosol exposure system, A549 cells, Titanium oxide nanoparticle toxicity, Barium sulfate nanoparticle toxicity, Air–liquid interface

## Abstract

We present a novel lung aerosol exposure system named MALIES (modular air–liquid interface exposure system), which allows three-dimensional cultivation of lung epithelial cells in alveolar-like scaffolds (MatriGrids^®^) and exposure to nanoparticle aerosols. MALIES consists of multiple modular units for aerosol generation, and can be rapidly assembled and commissioned. The MALIES system was proven for its ability to reliably produce a dose-dependent toxicity in A549 cells using CuSO_4_ aerosol. Cytotoxic effects of BaSO_4_- and TiO_2_-nanoparticles were investigated using MALIES with the human lung tumor cell line A549 cultured at the *air–liquid interface*. Experiments with concentrations of up to 5.93 × 10^5^ (BaSO_4_) and 1.49 × 10^6^ (TiO_2_) particles/cm^3^, resulting in deposited masses of up to 26.6 and 74.0 µg/cm^2^ were performed using two identical aerosol exposure systems in two different laboratories. LDH, resazurin reduction and total glutathione were measured. A549 cells grown on MatriGrids^®^ form a ZO-1- and E-Cadherin-positive epithelial barrier and produce mucin and surfactant protein. BaSO_4_-NP in a deposited mass of up to 26.6 µg/cm^2^ resulted in mild, reversible damage (~ 10% decrease in viability) to lung epithelium 24 h after exposure. TiO_2_-NP in a deposited mass of up to 74.0 µg/cm^2^ did not induce any cytotoxicity in A549 cells 24 h and 72 h after exposure, with the exception of a 1.7 fold increase in the low exposure group in laboratory 1. These results are consistent with previous studies showing no significant damage to lung epithelium by short-term treatment with low concentrations of nanoscale BaSO_4_ and TiO_2_ in in vitro experiments.

**Supplementary Information:**

The online version contains supplementary material available at 10.1007/s00204-023-03673-3.

## Introduction

Every day, humans are exposed to ambient air, which contains air pollutants such as gaseous ozone, carbon monoxide and nitrous oxide, but also cigarette smoke, particulate matter, various types of allergens, and increasingly nanomaterials/nanoparticles; all of which are potentially hazardous to human health. These nanomaterials, because of their very small size (by definition 50% of the particle number-based size distribution) are between 1 and 100 nm in at least one dimension (2011, 2022), could possibly enter human cells and could have unexpected adverse effects on the lungs and other organ systems (Bonner 2010). Like the skin, the lungs come into direct contact with nanoscale materials, which can lead to lung inflammation, oxidative stress, and lung dysfunction (You and Bonner 2020). Because of its unique morphology and multiple protective mechanisms, the lung can usually mitigate major damage to the alveolar epithelium from inhaled chemicals and repair damage that has occurred (Donaldson and Tran 2002; Monteiller et al. 2007; Pauluhn 2009). Nevertheless, some studies show, that chronic exposure to nanoscale materials may lead to long-term damage of the respiratory system as well as systemic immune effects as shown for the effect of carbon black nanoparticles in rats (Chu et al. 2019).

The lung alveoli are lined with a very dense layer of epithelial cells (type I and type II pneumocytes). Type I epithelial cells cover about 90% of the total surface area and are the primary site of gas exchange to the capillary endothelial cells of the vasculature (Overgaard et al. 2012). Type II pneumocytes are with 10% underrepresented in the alveoli but still have important functions: they produce surfactant proteins to reduce surface tension in the lung and mucins (MUC5AC, MUC5B) that are secreted and polymerize to form mucus or are cell surface associated (MUC1, 4, 16 and 20) (*Ma, 2018*). Alveolar epithelial cells are connected to each other by tight and adherent junctions resulting in the formation of a strong alveolar-epithelial barrier (Overgaard et al. 2012) which together with the pulmonary endothelial cells form the epithelial-endothelial barrier in the lung.

A large number of in vitro studies examined the cellular effects of nanomaterials on respiratory epithelial cells (Foldbjerg et al. 2011; Guadagnini et al. 2015; Heng et al. 2011; Herzog et al. 2013; Hsiao and Huang 2011; Kim et al. 2011; Park et al. 2008; Sayes et al. 2007; Yu et al. 2013; Zhang et al. 2011) in addition to in vivo inhalation or instillation studies performed with animals, mostly rats (Donaldson et al. 2008; Kim et al. 2011; Klein et al. 2012; Landsiedel et al. 2014a; Molina et al. 2019; Pauluhn 2010; Song et al. 2013; Sung et al. 2009). Comparability among these in vitro studies tends to be poor due to the use of different laboratory-specific protocols for nanomaterial preparation, cultivation- and incubation times (Landsiedel et al. 2014b). In addition, the translation of the results to the real in vivo situation, and thus the predictability of these in vitro models for toxicity to the respiratory tract, is questionable. Moreover, most of these studies used non-physiological cultivation conditions, e.g., submerged cultures for the analysis of nanoparticle toxicity to respiratory epithelial cells (Gliga et al. 2014; Hsiao and Huang 2011; Remzova et al. 2019). However, these studies do not reflect the real in vivo situation, since due to the given anatomy of the lung, aerosolized particles (e.g., inhaled nanoparticles) reach bronchial and subsequently alveolar epithelial cells together with the respiratory air at the *air–liquid interface* (ALI), where gas exchange takes place. For this reason, there has been an evolution in recent years from the earlier rather simple in vitro assays to complex lung models with multiple cell types/co-cultures (Jing et al. 2015; Klein et al. 2013; Rothen-Rutishauser et al. 2005), ALI cultivation with cell lines (Braakhuis et al. 2015; Zscheppang et al. 2018) or primary cells (Kao et al. 2005; Thai et al. 2005; Zscheppang et al. 2018), commercial ALI models such as EpiAirway (MatTek Corporation, MA, USA), OncoCilAir™ and MucilAir™ (Epithelix Sarl, Switzerland) with ALI cultured primary cells from healthy and diseased donors, and finally lung aerosol exposure devices, which are mostly commercially available (VITROCELL™, Vitrocell Systems GmbH, Waldkirch, Germany; CULTEX, Cultex Laboratories GmbH, Hannover, Germany) in combination with ALI cultured mono- and cocultures (Aufderheide and Mohr 2000; Barosova et al. 2020; Braakhuis et al. 2020; Gervelas et al. 2007; Hufnagel et al. 2020; Niwa et al. 2007). By approximating real in vivo conditions, aerosolized ALI lung models are considered as an approach to implement the "3-R principle": replace, reduce and refine the use of animals in lung toxicity studies (Russell and Burch 1992; Upadhyay and Palmberg 2018).

While the Cloud system of the Vitrocell™ exposure device uses nebulization and gravitational settling for particle deposition, other in vitro aerosol exposure systems work with electrostatic precipitation for the exposure of ALI cultivated cells (de Bruijne et al. 2009; Frijns et al. 2017; Mülhopt et al. 2016). Similar to Vitrocell, the ALICE (air–liquid interface cell exposure) system generates particle-droplet clouds by nebulization which sediment on the ALI cultures (Lenz et al. 2009). ALICE enables for uniform and dose-controlled deposition of nanoparticle suspensions; however, loading in suspension form may affect the physicochemical properties of the nanoparticles (Meaney et al. 2002). Therefore, reliable experiments should use dry, aerosolized nanoparticles to lower such aggregation effects (Duret et al. 2012). Such a device that disperses dried nanoparticles onto ALI cultures was developed by Ji et al. (2017). The Xpose*ALI*^®^ exposure model, is able to aerosolize nanoparticles in a powder chamber by compressed air of 100–140 bars. Generated aerosol is then pulled from the holding chamber into the exposure manifold at a main flow rate of 90 ml/min, where triplicate model inserts are exposed at the same time. Xpose*ALI*^®^ system (Inhalation Sciences, Huddinge, Sweden) is commercially available with flexible aerosol sources: beside powder also nebulizer can be used. But wet aerosols make it difficult to characterize exposure conditions, as most techniques applied to acquire size-distributions of ultra-fine aerosols—especially in real-time—cannot discriminate water droplets from other particles, which can lead to over-estimation of exposure doses. Additionally, nanoparticles tend to form agglomerates or aggregates in wet atmospheres. Recently, a novel Dosimetric Aerosol in vitro inhalation device (DAVID) was demonstrated (Ward et al. 2020), which was used for the investigation of the effect of ultrafine particles from welded fume on lung epithel by water-based condensation. This system delivers substantial doses in minutes (≥ 100 µg/cm^2^) compared to earlier exposure systems that require hours for sufficient dose. Newest aerosol exposure systems work with breathing-like 3D cyclic stretch chips (AX lung chip, Alveolix AG; (Sengupta et al. 2022) and actively breathing exposure systems (Steiner et al. 2020).

Most studies with the described aerosol exposure devices use transwell inserts with a planar, permeable foil, on which either monocultures or complex cocultures are grown under ALI conditions (Barosova et al. 2020; Hufnagel et al. 2020; Klein et al. 2013), but lack the typical 3D morphology of the alveolus.

We present a novel in vitro aerosol exposure system with which nanoparticle toxicity can be investigated after generation of nanoparticle aerosols by nebulization followed by dehumidification of the aerosol with a diffusion dryer with silica gel desiccant. Together with the 3D polycarbonate scaffold (MatriGrid^®^) which, by its special cavity morphology, keeps the cultured lung cells humid during aerosol exposure, it represents a simple lung model (Mai et al. 2014, 2017) composed of several modular units that can be easily assembled and used for aerosol exposure experiments.

Nanoscale barium sulfate (BaSO_4_) and titanium dioxide (TiO_2_) were selected as test substances for the MALIES. The data available on BaSO_4_ are comparatively limited in contrast to other nanomaterials with high production volumes such as titanium dioxide, silicon dioxide or zinc oxide despite its widespread use and therefore the regulatory assessment is less robust. BaSO_4_ has a wide range of applications in building materials as well as in consumer products and thus has direct contact with humans. BaSO_4_ is a component of high-performance epoxy resins, in paints and as a filler in the paper/plastics industry (Petrova et al. 2008) as well as in medical technology as a contrast agent and implants such as catheters and in bone cement (Aninwene et al. 2013; Ricker et al. 2008). Existing data on the short- and long-term health effects of BaSO_4_ nanoparticles on the lung are still incomplete. In vivo (rats), BaSO_4_ showed no toxic effects after inhalation (5 days) or instillation (28 days). After 13 weeks of inhalation, there was a slight increase in inflammatory markers in the lungs (Konduru et al. 2014). Barium from BaSO_4_ NPs is distributed in the body and is excreted via urine and faeces (Konduru et al. 2014; Molina et al. 2019). Furthermore, (Molina et al. 2019) and (Konduru et al. 2014) found that barium from BaSO_4_-NPs is mainly translocated from the lungs after dissolution. The barium ions are then mainly taken up in the bones and other organs. So far, only a few in vitro data on nanoscale BaSO_4_ have been collected. (Kroll et al. 2011) showed that after testing on 10 cell lines, BaSO_4_ significantly inhibited metabolic activity only in fibroblasts.

Due to the good data situation and the effects of nanoscale titanium dioxide (TiO_2_) proven in many *in-vitro* studies, TiO_2_ was selected as a second test substance. In addition, it is mechanistically suitable for the research project, since its effects, like those of BaSO_4_, are not mediated via released ions (Maynard and Kuempel 2005; Oberdorster 2002; Tran et al. 2000). TiO_2_ is often used as a positive control in particle toxicology, as many studies have already demonstrated toxic effects. It has been shown that when administered intraperitoneally in mice and rats, the particles cause damage to the liver and kidneys (Abbasi-Oshaghi et al. 2019; Alarifi et al. 2013; Li et al. 2010). Lung damage and tumors have also been observed in rats after inhalative administration of TiO_2_ (Baisch et al. 2014; Bermudez et al. 2004).(Boland et al. 2014) clarified the cellular mechanism of TiO_2_ toxicity. The particles enter the cell via lysosomes which are damaged by the particles. Released hydrolases activate the inflammasome and lead to apoptosis (Boland et al. 2014).

## Materials and methods

### Chemicals and reagents

DMEM, PBS (sterile), sodium pyruvate, penicillin/streptomycin, protein standard, bicinchoninic acid solution and copper (II) sulfate solution for total protein determination were purchased from Sigma-Aldrich (Taufkirchen, Germany); fetal bovine serum and glutamine were from Biochrom (Berlin, Germany). Resazurin was from Sigma Aldrich (Taufkirchen, Germany) and Alamar blue kit from BioRad (Feldkirchen, Germany) (BUF 012-B). LDH-Kit was purchased from Sigma-Aldrich (MAK066-1KT) with an additional standard from Cayman-Chemical (Michigan, USA). For the GSH enzymatic recycling method potassium dihydrogen orthophosphate, dipotassium hydrogen orthophosphate and Triton X-100 were aquired from Carl Roth (Karlsruhe, Germany). Sulfosalicylic acid was purchased from Sigma-Aldrich (Taufkirchen, Germany). DTNB, ß-NADPH, Glutathione reductase and GSH were purchased from Merck (Darmstadt, Germany). Diethiothreitol and Monobrombimane were from Sigma Aldrich (Taufkirchen, Germany).

### Cell culture

Human lung carcinoma A549 cells representing the alveolar type II phenotype (Lieber et al. 1976) were obtained from ATCC (Manassas, USA). Cells were cultured in tissue culture flasks in DMEM supplemented with 10% fetal bovine serum (FBS), 1% sodium pyruvate, 2% glutamine and 100 U/ml penicillin/100 µg/ml streptomycin (Pen/Strep) at 37 °C in a cell incubator at 95% relative humidity and 5% CO_2_. Medium was changed every 2–3 days. A549 cells were split according to a standard protocol.

### 3D cell carrier MatriGrid^®^ and semi-active insert system

**Fig. 1:**
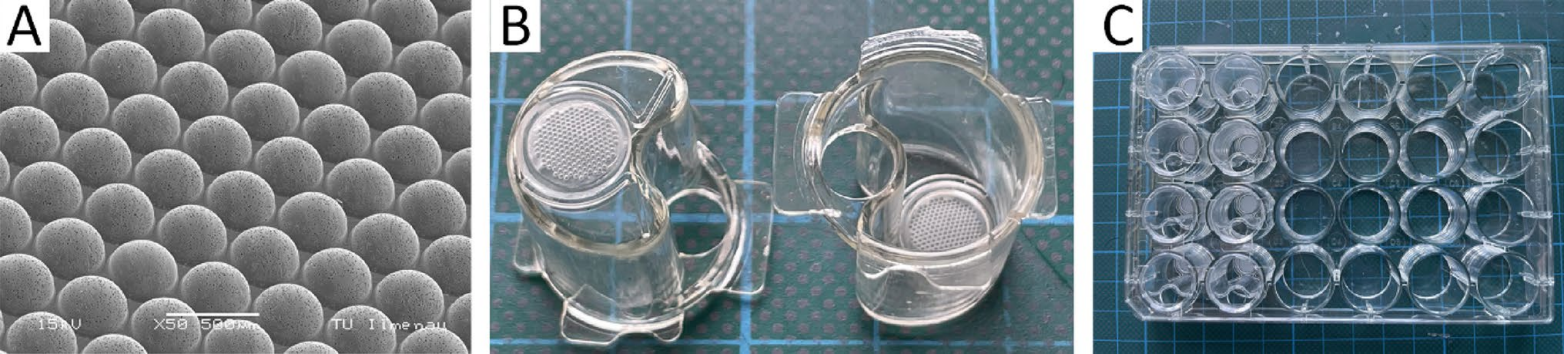
3D cell carrier MatriGrid^®^ and semi-active insert system. **A** Scanning electron microscopy image of apical MatriGrid^®^ direction with porous cavities. **B** MatriGrid^®^s welded into the semi-active insert systems. **C** 24 well plate filled with semi-active insert systems

Three-dimensional cultivation of A549 cells was carried out in porous polycarbonate scaffolds named MatriGrid^®^ (Fig. 1a), whose shape and size are comparable to the morphology of the lung alveoli. The scaffold consists of a circular 50-micron thick biocompatible polycarbonate (PC) foil with a micro structured seeding area of 26.06 mm^2^ wherein 187 microcavities for cell cultivation are formed. Fabrication and quality control of the MatriGrid^®^-scaffolds have been previously described in Borowiec et al. (2015). A combined micro thermoforming- and etching technique was used to treat the porous PC foil in such a way that pores were exclusively present in the area of the microcavities (Hampl et al. 2012). Pores are necessary for the nutrient supply of cultivated lung cells inside MatriGrids^®^. MatriGrids^®^ were embedded in semi-active insert systems (Fig. 1b) to enable cultivation under *air liquid interface* (ALI) conditions. The insert system is divided into two separated compartments whereby the cells can be cultured under ALI condition due to the precisely adjusted addition of medium in the lower compartment. Thus, cells on the apical scaffold side are supplied with medium through the pores from the lower compartment and are in contact with air at the top. Semiactive insert systems are placed in 24-well plates (Greiner Bio-One, Frickenhausen, Germany) (Fig. 1c) for ALI culturing of A549 cells.

### Modular air–liquid interface aerosol exposure system (MALIES)

Commercially available components were selected as far as possible for the experimental setup. A digital controller was used as the mass flow controller (5200 series, TSI, Aachen, Germany). The aerosol was dried using a diffusion dryer filled with silica gel (DDU 570/H, Topas, Dresden, Germany). The manufacturer uses it for similar experimental setups and thus achieves drying of corresponding volume flows. The aerosol flow was distributed with antistatic polyurethane hoses (PUN 12 × 10 Antistat, Landefeld Druckluft und Hydraulik, Kassel-Industriepark, Germany). A Scanning Mobility Particle Sizer (SMPS, NanoScan SMPS 3910), an Optical Particle Sizer (OPS, Optical Particle Sizer 3330), a Condensation Particle Counter (CPC, CPC Mod. 3775, all from TSI, Aachen, Germany) and an Aerodynamic Aerosol Classifier (AAC) (AAC, Cambustion, Cambridge, UK) were used to measure the nanoparticles. To achieve the desired flow distribution in the channels, the flow in each channel was controlled either manually, with a variable area flow meter, or automatically by the computer-controlled, commercially available Cell Culture Exposure System 3430 (TSE Systems GmbH, Bad Homburg, Germany). The commercially available exposure system comprises two mass-flow controllers (MFC) for flow in and flow out and one additional MFC for CO_2_ per channel. Exposure conditions, like flows and system pressure, were controlled by DACO-software. (TSE Systems GmbH, Bad Homburg, Germany).

Within the design of the modular exposure system, all edges were rounded with a smooth transition and no undercuts were created thus ensuring a uniform and reproducible exposure flow to the lung cell cultures over a long period. The design was repeatedly checked and optimized by simulation. The components were machined by turning and milling with subsequent surface treatment. The material chosen was 1.4571 (V4A). CNC milling was done by a local manufacturer.

To protect the cell cultures from cooling down during long exposure times, the micro titer plate with the exposure module was set up on a hot plate (VWR International GmbH, Darmstadt, Germany) at 37 °C.

A binary tree structure with constant flow velocity was calculated to distribute the aerosol without disturbance into four equal channels. After the distribution of the aerosol to the cell cultures, all channels are merged into a central collection channel leading to a filtered outlet. The module was designed as a mirror-symmetrical component with a central dividing plane, which allowed very good accessibility to all contours. All components are detachable by means of screw connections. All inner contours could thus be produced with a low roughness depth of Ra < 0.8 µm. Due to the division, the half channels have a good accessibility for cleaning and decontamination. A shaped seal that follows the contour of the insert system allows each compartment to be shielded from external influences. By adjusting the shaped seal and nozzle, the exposure module can be adapted to different cell cultures in different carriers.

Prior to each experiment, the exposure unit was sterilized in a disassembled state with 70% ethanol for 30 min and dried overnight in a laminar flow cabinet.

### Simulation of particle flow and investigation of particle distribution with micro particles

To investigate whether the MALIES distributes particle flow evenly over the four channels, a simulation model was created and measurements with fluorescent micro particles (FluoSpheres^®^-Carboxylated-Modified Microspheres, F8795, Thermofisher, Waltham, USA) were performed in preliminary experiments. The particles with a size of 40 nm were nebulized for 10 min in a final concentration of 50 µg/ml in a Pariboy nebulizer at 0.9 l/min. After passing the diffusion dryer, the air-particle mixture flowed into the exposure module and was finally deposited on impermeable membranes in a 24-well plate. Deposited particles were then rinsed with 100 µl A. dest and transferred to separate 1.5 ml Eppendorf tubes after resuspending several times. The measurement of particle fluorescence took place on a plate reader (SpectraMax M5, Molecular Devices), where the collected fluosphere suspensions of each membrane/well were measured in black 96-well plates. To determine the percentage distribution of the fluospheres by the exposure module, the fluorescence signal of a single insert was divided by the sum of the fluorescence of all inserts.

Simulation of flow was done using Ansys CFX. The flow in the calculation area can be considered as laminar. The model (exposure module) was meshed with tetrahedra (0.4 mm in size) and 10 inflation layers were added (first layer thickness 25 µm). The inlet port was imprinted with a flow rate of 0.9 l/min as velocity. The outlet was set as 0 Pa as the pressure outlet. In the evaluation, the mass flow of the individual channels was compared. Again, to determine the distribution, the mass flow from one channel was divided by the sum of all mass flows.

### Air–liquid interface (ALI) culturing of A549 cells in semi-active systems containing MatriGrids^®^

MatriGrids^®^ were sterilized in 100% ethanol for 15 min followed by incubation in 70% ethanol for 15 min and treatment in a descending ethanol series for deaeration. MatriGrids^®^ were washed with A. bidest and then transferred to well plates. 500 µl medium was added to the basal side of the MatriGrid^®^. 25 µl of a cell suspension containing 1 × 10^5^ A549 cells was seeded onto the apical side of the scaffolds. To ensure targeted seeding in microcavities the cells were given an adherence time of one hour at 37 °C and 5% CO_2_ in the cell incubator, 500 µl medium was subsequently added to the apical side of the MatriGrid^®^. MatriGrids^®^ with A549 cells were incubated for 24 h under submerged conditions (500 µl medium each on the apical and basal side of the MatriGrid^®^ in the insert), followed by incubation at *air–liquid interface* (ALI, 500 µl medium on the basal side) for 3–5 days.

### Generation of an exposure control with CuSO_4_ aerosol with MALIES (dose response experiment)

A549 cells were precultured in MatriGrids^®^ in ALI culture for 72 h. Subsequently, A549 cells were exposed with the MALIES with clean air (negative control) and CuSO_4_ aerosol in increasing concentrations (from 1 g/l, 2 g/l, 5 g/l, 10 g/l, 20 g/l, 30 g/l and 40 g/l CuSO_4_-H_2_O-solution) for one hour on a hot plate set to 37 °C in a laminar flow cabinet. During exposure cells were incubated in medium supplemented with 20 mM HEPES buffer for maintenance of pH. After exposure cells were shifted to fresh medium without serum and 24 h post incubation the metabolic activity of the cells were determined with the resazurin assay. The resazurin assay and analysis of data were performed like described below.

### Copper (Cu) colorimetric assay

To quantitatively measure the amount of copper deposited during exposure, inserts with impermeable membranes were used. These were exposed to CuSO_4_ alongside the A549 in MatriGrids^®^ using the MALIES exposure module for one hour. The copper deposited on the membranes was dissolved in 20 µl of Millipore water and measured using a commercially available calorimetric test kit (Elabscience; 41528-96). Samples were diluted with Millipore water (1:5; 1:20; 1:100; 1:200) to match the calibration curve of the kit of 5–60 µmol/l. The assay used is based on the reaction of copper ions with 3,5-DiBr-PAESA, forming a violet complex that is detected at 580 nm.

### Nanoparticle preparation and aerosol exposure conditions

BaSO_4_-nanoparticles were from Huntsman (Salt Lake City, USA) and Aeroxide^®^ P90 TiO_2_-nanoparticles from Evonik (Essen, Germany). BaSO_4_ nanoparticles had a size of 40 nm and TiO_2_ nanoparticles of 17 nm. Bertolotti et al. (2020) analyzed the structure, morphology, and faceting of Aeroxide^®^ P90 TiO_2_ nanoparticles and found that 13% were in the rutile phase and 87% were in the more photoactive anatase phase. Due to their smaller size compared to the also commonly used Aeroxide^®^ P25 TiO_2_ nanoparticles and the associated larger surface area, their photocatalytic activity is higher.

Dry nanoparticles were suspended in A. bidest to a stock concentration of 10 g/l and ultrasonicated. Stock suspensions were further diluted with A. bidest to working suspensions. Different concentrations of suspension concentrations were analyzed in terms of the nanoparticle concentration in air with SMPS. Due to the detection range of the SMPS and clogging of the aerosol generator, the maximum achievable suspension concentration was 0.9 g/l. Therefore, working solutions with nanoparticle concentrations of 0.1 g/l and 0.9 g/l were determined for the final experiments. The working solutions were ultrasonicated again with the following settings (BaSO_4_-NPs: three minutes; application of energy ~ 15 kJ; TiO_2_-NPs: one minute; application of energy ~ 5 kJ).

After culturing A549 cells under ALI condition in MatriGrids^®^, the respecting well plate with cells was placed on a hot plate set to 37 °C under a laminar flow cabinet. Cells in MatriGrids^®^ were exposed to clean air (negative control) or BaSO_4_- and TiO_2_-NP aerosol with the suspension concentrations of 0.1 g/l and 0.9 g/l (corresponding aerosol concentrations are to be found in Table 4) for 1 h with the MALIES. During simultaneous exposure of four wells with the exposure unit, the other wells in the 24-well were covered with an appropriate lid.

Due to the existing measuring equipment and devices at the different institutions, the exposure conditions of laboratory 1 (TU Ilmenau) and laboratory 2 (Martin Luther University Halle) had to be adapted. For the maintenance of pH of the cell culture media during nanoparticle exposure, laboratory 1 supplemented the medium with 20 mM HEPES buffer while laboratory 2 used CO_2_ gas perfusion. In both cases analysis of pH revealed pH levels between 7.3 and 7.6.

### MUC5-AC/SP-C and ZO-1/E-cadherin immunofluorescence

After 72 h of ALI-culturing, A549 cells grown in inserts on MatriGrids^®^ were washed twice with phosphate buffered saline (PBS) and subsequently fixed for 15 min with 4% paraformaldehyde in PBS. Cells were permeabilized with 0.25% Triton in PBS for 15 min and blocked with 1% BSA in PBS for 30 min. Cells were incubated with the following antibodies overnight: rabbit anti human Mucin (#61193, Cell Signaling Technology Europe, Leiden, Netherlands), mouse anti human SP-C (sc-518029; Santa Cruz Biotechnology), mouse anti human ZO-1 (610967; BD transduction laboratories, Sarl, Switzerland) and rabbit anti human E-Cadherin (# 3195, Cell Signaling Technology Europe, Leiden, Netherlands) followed by incubation with species-dependent secondary antibodies: Alexa Fluor 488 labeled goat anti-rabbit antibody and Alexa Fluor 647 labeled goat anti-mouse antibody (Thermofisher, Darmstadt, Germany). Cells on coverslips were mounted in FluoromountG containing DAPI (00-4959-52, Thermofisher Scientific, Darmstadt, Germany). Images were captured with an OLYMPUS laser scanning microscope FV1000 (Olympus, Germany).

### SEM imaging

Morphology of A549 cells cultured under ALI for 72 h was examined with scanning electron microscopy (SEM). Cells were fixed using 2.5% glutaraldehyde at 4 °C for 1 h and subsequently washed two times with A. bidest. After drying the samples, they were sputtered with a thin platinum layer and examined by scanning electron microscopy (SEM Hitachi S 4800-II, Hitachi High-Technologies Europe GmbH).

### TEM imaging/particle deposition

To have a second method to characterize the aerosols we exposed copper grids to the test-aerosols (0.1 g/l-groups and controls: 10 min; 0.9 g/l-groups: 5 min). Subsequently, grids were analysed without fixation using an EM 900 electron microscope (Zeiss Microscopy GmbH, Jena, Germany) with an acceleration-voltage of 80 kV. Images were recorded using a Variospeed SSCCD Camera SM-1 k-120 (TRS, Moorenweis, Germany). Particles were counted and measured in overview screens. Subsequently particle numbers per 5 or 10 min and area of view were calculated. The volume and the mass of the particles, respectively, was calculated via the diameter. Finally, all masses were summed and extrapolated to the area of the MatriGrid^®^.

### Resazurin/alamar blue assay

After the appropriate post exposure times (24 h and 72 h) after nanoparticle exposure, cells were incubated with 10% Alamar Blue™ solution containing resazurin (laboratory 1) or with self-made resazurin-solution (11 mg/l in PBS) (laboratory 2) for 1 h at 37 °C. In viable cells resazurin is reduced to resorufin by mitochondrial enzymes. The concentration of resorufin generated by A549 cells was determined by fluorescence spectrometry (ex: 530 nm; em: 590 nm) in a plate reader (Spectramax, Molecular Devices, San Jose, USA; laboratory 1 and Tecan Genios, Tecan, Männedorf, Switzerland; laboratory 2). Relative viability was calculated by normalization of measured values from nanoparticle exposed samples to the value of air-exposed control which was set to 100%.

### LDH assay

LDH assays were performed 24 h and 72 h after nanoparticle exposure.

*Laboratory 1*: A commercially available LDH-Assay kit (Sigma Aldrich, Taufkirchen, Germany; MAK066-1KT) was used and the instructions of the provider were followed. LDH concentrations were determined by comparison with a concurrently generated calibration curve in the range of 0–6.25 mM. In this assay, LDH reduces NAD to NADH, which is specifically detected by this colorimetric assay at 450 nm. A 100% Triton X-100 LDH release control was included.

*Laboratory 2*: Medium supernatants after nanoparticle exposure were transferred to a 96-well plate. For the enzyme reaction 0.4 mM NADH reaction mix and 2 mM Na-pyruvate buffer was added. The extinction was measured at 340 nm with a plate reader (Tecan Genios, Tecan, Männedorf, Switzerland). A 100% LDH release control prepared by lysis with 10% triton detergent was included.

In both laboratories LDH values measured from nanoparticle exposed A549 cells were normalized to the values of air-exposed control which was set to 1 (relative LDH amount).

### Determination of total intracellular glutathione content

*Laboratory 1*: Total Glutathione levels (GSH + GSSG) were determined according to the method of Rahman et al. (2006). Briefly, the cells of one MatriGrid^®^ were lysed by freezing (− 80 °C) in extraction buffer containing sulfosalicylic acid, followed by thawing on ice and repeated sonication and vortexing. After sonication the cells were frozen at − 80 °C once again, thawed on ice and centrifuged. Supernatant was transferred into pre-chilled Eppendorf tubes. The GSH levels in the supernatant were spectroscopically determined using the enzymatic recycling method with DTNB [Ellmans reagent = 5,5′-dithiobis-(2-nitrobenzoic acid)], where one molecule GSH reacts with DTNB resulting in one molecule TNB chromophore and one molecule Glutathion-TNB. The total protein content of the samples was determined from cell lysates via a bicinchoninic acid assay.

*Laboratory 2*: Two MatriGrids^®^ per condition were lysed with 250 μl 0.1 N HCl, pooled and frozen at − 80 °C. After thawing, samples were reduced with DTT (diethiothreitol) and subsequently derivatized with monobromo-bimane. The total amount of glutathione (GSH + GSSG) was determined by HPLC with fluorescence detection (ex: 380 nm; em: 480 nm). Conditions for chromatography: stationary phase: Chromolith Performance RP-18e, 5–4.6 mm (Merck, Darmstadt, Germany); mobile phase: solvent A: 2% methanol/98% water/0.23% acetic acid (pH 4.3); solvent B: 90% methanol/10% water/0.25% acetic acid (pH 3.9). Analysis was conducted using the following gradient: 0–5 min: 97% A, 3% B; 5–9 min: 93% A, 7% B; 9–10 min: 92% A, 8% B; 10–14 min: 0% A, 100% B; 14–15 min: 0% A, 100% B; 15–18 min: 97% A, 3% B. Injection volume was 30 μl.

In both laboratories total glutathione values measured from nanoparticle exposed A549 cells were normalized to the values of air-exposed control which was set to 1 (relative glutathione amount).

### Statistical analysis

Mean values and standard deviations were calculated from at least three independent experiments. Significances (p-values) were estimated by Friedman-ANOVA and Bonferroni correction.

## Results:

### Setup of the modular air–liquid interface system (MALIES)

**Fig. 2:**
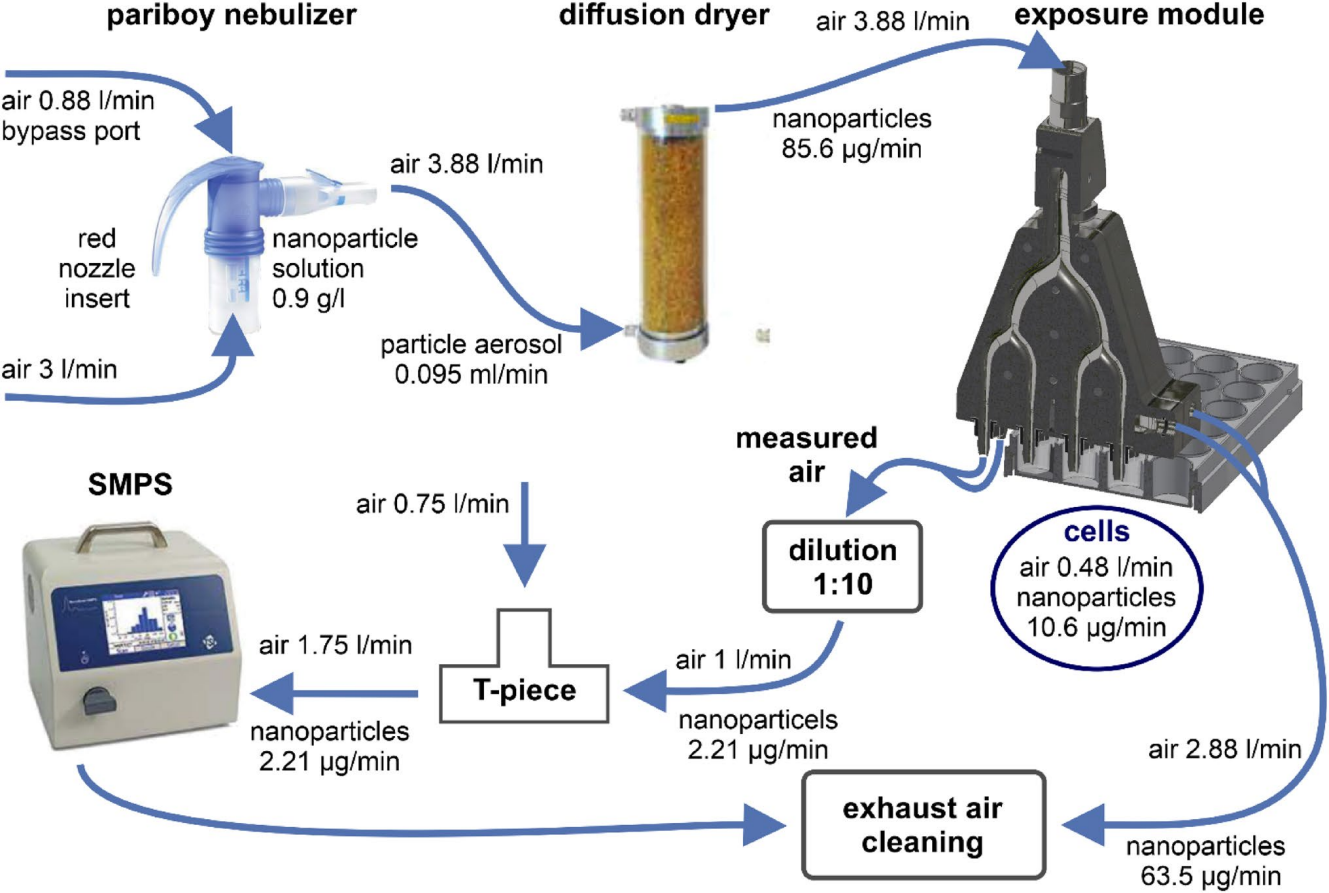
Setup of the MALIES with flow diagram and volumetric flows. MALIES consists of a Pariboy unit for nebulization, a diffusion dryer with silica gel for dehumidification and an exposure module for uniform exposure of nanoparticle aerosols on ALI-cultured lung cells on semi active systems containing MatriGrid^®^-alveolar scaffolds. Incoming aerosol is separated into four channels, three of which are used to expose the lung cells and the other one measures particle size and amount with SMPS. The arrows indicate the path of the flow and the expected or set flow rates of the measurement setup. It should be noted that the particle flow is a calculated value, which is lower in the real system

The MALIES was designed in such a way that several modular units for aerosol generation, conditioning, distribution and exposure form a functional, simple overall system that can be quickly set up and put into operation. Figure 2 shows the experimental setup with integrated flow diagram and volumetric flows.

For aerosol generation, we selected a commercially available PARI BOY SX Compressor combined with a PARI LC SPRINT STAR nebulizer (red nozzle insert, Pari GmbH, Starnberg, Germany), which is normally applied in inhalation therapy and especially designed for the generation of deep lung aerosols. After aerosol generation from the nanoparticle suspension, the aerosol stream passes through a diffusion dryer (TOPAS DDU 570/H, TOPAS GmbH, Dresden, Germany) with silica gel resulting in the formation of dehumidified aerosolized nanoparticles (Fig. 2). Distribution of the aerosol flow occurs with antistatic polyurethane tubes. Inside the exposure module (Fig. 2), the aerosol is evenly divided into four (eight) channels, three (six) of which are used for exposure of ALI-cultured A549 cells in MatriGrids^®^ and one serves as measurement channel for SMPS (scanning mobility particle sizer) and OPS (optical particle sizer) or CPMA (Centrifugal Particle Mass Analyzer). Exposure of control cells with air (without nanoparticles) occurred in separate experiments with a similar exposure module.

The aerosol that passed through the cell cultures, is collected, filtered and released into the exhaust air of the sterile workbench. To avoid overloading of the SMPS during measurement, a 1:10 dilution stage (TSI 3332, TSI GmbH, Aachen, Germany) was integrated in the measurement channel.

The exposure module has a simple and robust design, is cleanable, sterilizable and fits on standard micro titer plates (24-well multi well plates from Greiner, Frickenhausen, Germany). The MALIES is a completely closed system to avoid contamination of the environment with nanoparticles and simultaneously provide a sterile environment for the cell cultures.

### Aerosolized particle distribution-simulation and real distribution

**Fig. 3:**
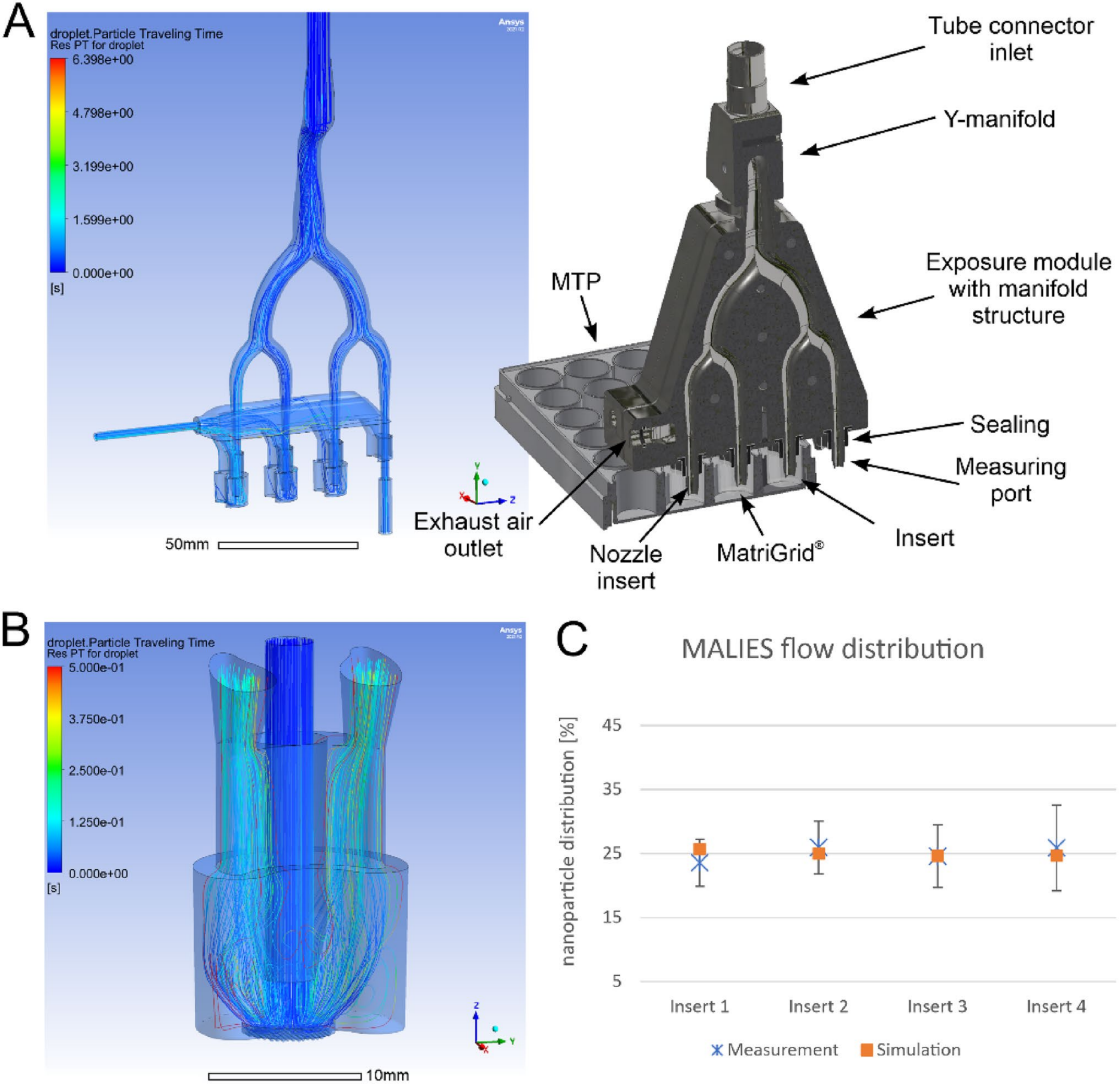
Simulation and real distribution of particles: **A** (left) Particle paths and residence times in the exposure module. **A** (right) Structure and components of the exposure module. **B** Detailed simulation of the particle paths in the area of the semi-active insert system. **C** Comparison of simulation and real measurement of Fluosphere^®^ particle distribution on the four channels by the exposure unit

During cell exposure experiments, two exposure modules were placed on 24-well plates containing semi active insert systems with MatriGrids^®^. Therefore, the flow behavior within the exposure module was simulated by using two channels instead of one as shown in Fig. 3a. Here, 3.88 l/min were provided at the inlet port and 0.5 l/min were used as flow out at each of the two measurement ports. The results of the simulation show that the mass flows of the individual channels deviate less than 3% from the nominal mass flow. Furthermore, it can be seen that there is only a small amount of vortex formation in the area of the nozzle or MatriGrid^®^ (Fig. 3b).

To determine the aerosol distribution through the exposure module of MALIES, fluorescent microparticles (Fluospheres^®^) with a size of 40 nm were used and aerosolized through 4 channels. The aerosol generation and experimental procedure are described in materials and methods. The measured and calculated percentages of fluorescent microparticles over four channels showed an almost uniform distribution of particle flow as shown in the diagram (Fig. 3c).

### Establishment of an exposure control (positive control) with MALIES

**Fig. 4:**
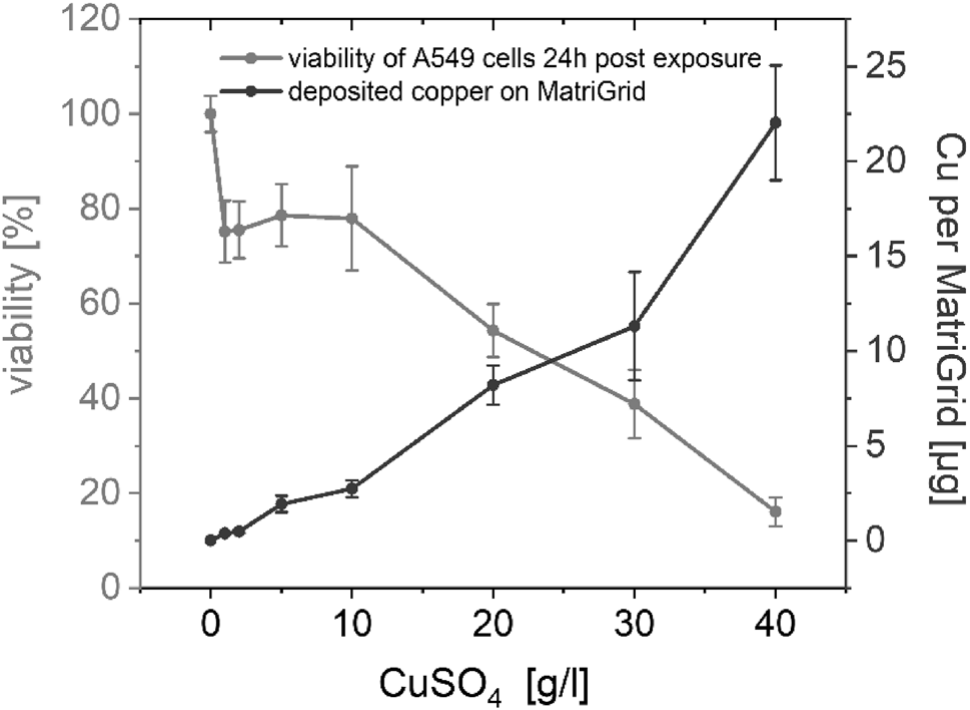
Dose-dependent reduction of viability of A549 cells and deposited mass of copper after aerosol exposure with a CuSO_4_ concentration gradient generated with MALIES. A549 cells were exposed for 1 h with increasing concentrations of CuSO_4_ aerosol and viability of cells was investigated with the resazurin assay 24 h post exposure. Concentration dependent copper deposition was investigated in parallel. Shown are the mean values and the standard errors of n = 3 experiments

MALIES was initially tested for its ability to produce a dose-dependent toxicity in lung cells upon aerosol exposure. The heavy metal copper II sulfate (CuSO_4_) is known to reduce lung cell viability due to oxidative stress (Chiou et al. 2023; Ritter et al. 2020). Therefore, we selected this compound to test the ability of MALIES to dose-dependently reduce the viability of ALI-precultured A549 cells after exposure to increasing concentrations of CuSO_4_ aerosol for one hour (up to 40 g/l suspension concentration). To correlate the toxicity with the copper concentration used, the deposition of copper on MatriGrids^®^ after exposure was determined in parallel with a colorimetric copper assay (Table 1). The experiments impressively show that MALIES produces a dose-dependent toxicity with CuSO_4_ aerosol in the lung cells 24 h after exposure (Fig. 4 and Table 1). This toxicity correlated well with the copper levels deposited on the surface of the MatriGrids^®^ (Fig. 4 and Table 1). The experimental setup tested here was used for all subsequent experiments with nanoparticle aerosol.

**Table 1:** Dose response experiments with CuSO_4_ aerosol: concentrations of CuSO_4_, deposition rate, deposited mass, deposited mass of copper per MatriGrid^®^ and dose-dependent reduction in viability of A549 cells measured 24 h post 1 h-exposure with MALIES are shown

Concentration CuSO_4_ (g/l)	Deposition rate (ng/min)	Deposited mass (µg/cm^2^)	Deposited mass per MatriGrid^®^ (µg)	Viability of A549 cells (%)
0	0.00	0.00	0.00	100.00 ± 3.82
1	6.30 ± 1.00	1.27 ± 0.20	0.38 ± 0.60	75.17 ± 6.53
2	8.10 ± 1.70	1.62 ± 0.33	0.49 ± 0.10	75.53 ± 6.01
5	32.20 ± 7.20	6.43 ± 1.44	1.93 ± 0.43	78.58 ± 6.57
10	45.50 ± 7.60	9.10 ± 1.52	2.73 ± 0.45	77.96 ± 11.01
20	136.70 ± 17.40	27.33 ± 3.47	8.20 ± 1.04	54.28 ± 5.61
30	188.50 ± 47.70	37.69 ± 9.54	11.31 ± 2.86	38.38 ± 7.21
40	367.20 ± 50.40	77.11 ± 19.34	22.03 ± 3.02	16.04 ± 3.01

Results are from *n* = 3 experiments, mean values ± SE

In all cell experiments with nanoparticles which used the resazurin assay as read out, the concentration of 30 g/l CuSO_4_ (corresponding to a deposited mass of 37.69 ± 9.54 µg/cm^2^) was chosen as the exposure/positive control, as this concentration reliably induced a decrease in metabolic activity of at least 50% (Fig. 4 and Table 1). Unfortunately, CuSO_4_ aerosol could not be used as an exposure control for cell experiments evaluated with the LDH assay, as the LDH enzyme is inhibited by copper ions and, therefore, interferes with the assay [own observation; (Han et al. 2011)]. For this type of viability assay a 100% release control, generated by treatment with 10% Triton, was chosen. 30 g/l CuSO_4_ has also been used successfully as a positive control in the HPLC assay for determination of glutathione.

### Determination of particle size distribution during nanoparticle exposure

**Fig. 5:**
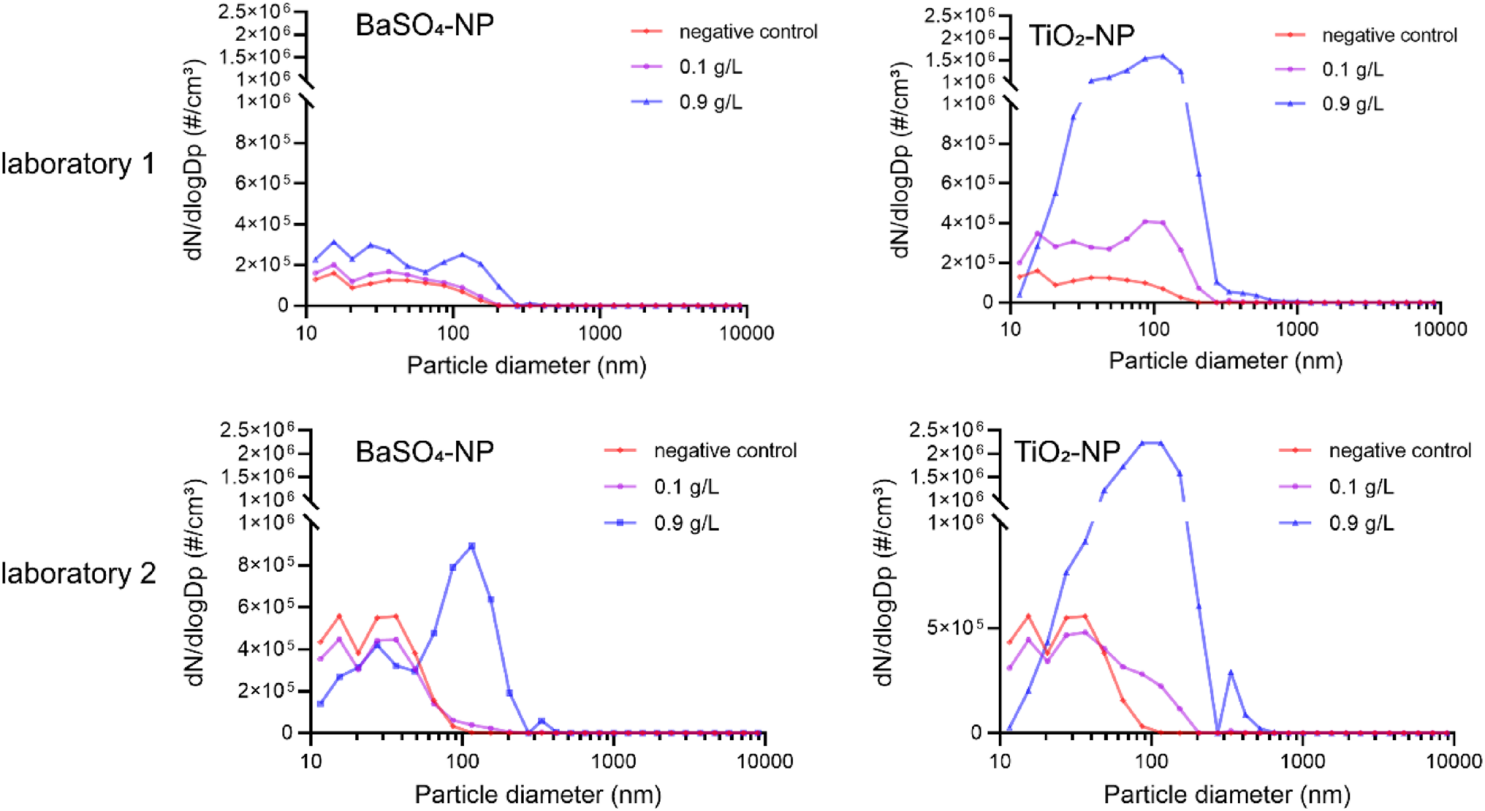
Size distribution of BaSO_4_-NP and TiO_2_-NP in aerosol exposure experiments. In both laboratories nanoparticle size distribution was measured with SMPS and OPS

During exposure with the MALIES device, nanoparticle size and amount was detected using SMPS (Table 2 and Fig. 5). In laboratory 1 the BaSO_4_-NP concentrations were 1.67 × 10^5^ and 3.08 × 10^5^ particles/cm^3^ and for TiO_2_-NP 3.96 × 10^5^ and 1.3 × 10^6^ particles/cm^3^ in the respective low and high exposure groups (Table 2). In laboratory 2 the BaSO_4_-NP concentrations were 3.21 × 10^5^ and 5.93 × 10^5^ particles/cm^3^ in the low (0.1 g/l) and high (0.9 g/l) exposure group; for TiO_2_-NP were the concentrations 4.23 × 10^5^ and 1.49 × 10^6^ particles/cm^3^ in the respective exposure group (Table 2). Thus, nearly similar particle amounts were detected using independent MALIES devices at different locations. The nanoparticle size distributions are shown in Fig. 5. While for TiO_2_-NPs the size distribution of nanoparticles was almost similar in the two laboratories, the size distribution of BaSO_4_-NPs differed. In laboratory 1 BaSO_4_-NP sizes ranged from 10 to 110 nm in the low (0.1 g/l) and from 10 to 120 nm with peak values of 20 nm and 100 nm in the high (0.9 g/l) exposure group. In contrast, in laboratory 2 BaSO_4_-NPs ranged from 10 to 100 nm with peak values of 30 nm in the low (0.1 g/l) and from 10 to 120 nm with peak values of 25 nm and 100 nm in the high (0.9 g/l) exposure group.

**Table 2:** Nanoparticle amount in the experiments measured with SMPS

Particle amount (particles/cm^3^)	Laboratory 1	Laboratory 2
BaSO_4_-NP (0.1 g/l)	1.67 × 10^5^	3.21 × 10^5^
BaSO_4_-NP (0.9 g/l)	3.08 × 10^5^	5.93 × 10^5^
TiO_2_-NP (0.1 g/l)	3.96 × 10^5^	4.23 × 10^5^
TiO_2_-NP (0.9 g/l)	1.3 × 10^6^	1.49 × 10^6^

The sizes of TiO_2_-NP in the low (0.1 g/l) exposure group showed a uniform distribution from 10 to 110 nm in both laboratories, while in the high (0.9 g/l) exposure group NPs with a size of 100 nm were predominant. In laboratory 2, the negative controls showed higher number values in the small particles than the low concentrations of both nanoparticle aerosols. This is due to remaining water droplets in the control aerosols. These already existing water droplets tend to form larger agglomerates with the small amount of nanoparticles added to the suspension, which is why the curve slides to the right rather than more particles being detected in the small channels.

With the help of these SMPS- and OPS measurements we also determined the actual mass flow for the nanoparticles under investigation. The Table 3 shows the measured and the maximum achievable value. The measurements show that at least 75% of the generated particles are deposited in the MALIES (between nebulizer and the SMPS). Nanoparticle aerosol concentrations exited through the outlet were calculated to be 0.29 mg/m^3^ BaSO_4_, 0.84 mg/m^3^ TiO_2_-NP in the low (0.1 g/l) and 3.15 mg/m^3^ and 10.90 mg/m^3^ in the high exposure group (0.9 g/l) using a total flow rate of 3.88 l/min (Table 4).

**Table 3:** Nanoparticle flow rate at measurement ports

particle flow rate (µg/min)	BaSO_4_-NP (0.1 g/l)	BaSO_4_-NP (0.9 g/l)	TiO_2_-NP (0.1 g/l)	TiO_2_-NP (0.9 g/l)
Maximum achievable value	2.45	22.05	2.45	22.05
SMPS/OPS	0.15 ± 0.05	1.51 ± 0.43	0.41 ± 0.12	5.23 ± 0.65

**Table 4:** Characterization of nanoparticle aerosols

	BaSO_4_-NP		TiO_2_-NP	
Concentration (g/l)	0.1	0.9	0.1	0.9
Deposition rate (ng/min) (minus negative control)	4.6	134.4	10.3	370.1
Concentration in air (mg/m^3^)	0.29	3.15	0.84	10.90
Deposited mass (µg/cm^2^)	0.9	26.6	2.1	74.0
Deposited mass per MatriGrid^®^ (µg)	0.3	8.1	0.6	22.2
Deposition efficiancy (%) (calculated from TEM)	3.2	8.9	2.6	7.1

### Nanoparticle deposition

To obtain an approximate deposition rate of the nanoparticles on the area of the MatriGrid^®^ (with A549 cells), TEM (Transmission electron microscopy) grids were placed on the MatriGrids^®^ and gassed for 5 or 10 min (with and without nanoparticles). Subsequently, TEM grids were analyzed by TEM by counting the particles in the measurement field. The Table 4 shows the data for each concentration investigated. The negative control (background) had a mean deposition rate of 1.2 ng/min and was subtracted from the values shown in the Table 4. It was calculated that during one hour exposure with nanoparticles (experimental condition during the cell exposure experiments) 0.3 µg BaSO_4_-NP and 0.6 µg TiO_2_-NP for the exposure concentration of 0.1 g/l and ~ 8.1 µg BaSO_4_-NP and 22.2 µg TiO_2_-NP for the exposure concentration of 0.9 g/l were finally deposited on the area of the grid/MatriGrid^®^ (Table 4). Corresponding deposited masses per cm^2^ are to be found in Table 4. Due to the use of the diffusion dryer, the deposition efficiency was rather low as expected. The positive/exposure control CuSO_4_ with a suspension concentration of 30 g/l showed comparable deposition rates as the nanoparticles (see Table 1).

### Characterization of MatriGrid^®^-cultured A549 type II pneumocytes

For NP-aerosol exposure experiments with MALIES we have used A549 lung type II pneumocytes as a cell culture model. Although they represent only the minor part of epithelial cells in the lung, they have important functions such as the production of mucus and surfactant proteins. A549 cells are easily cultivatable and allow obtaining fast and reproducible data.

**Fig. 6:**
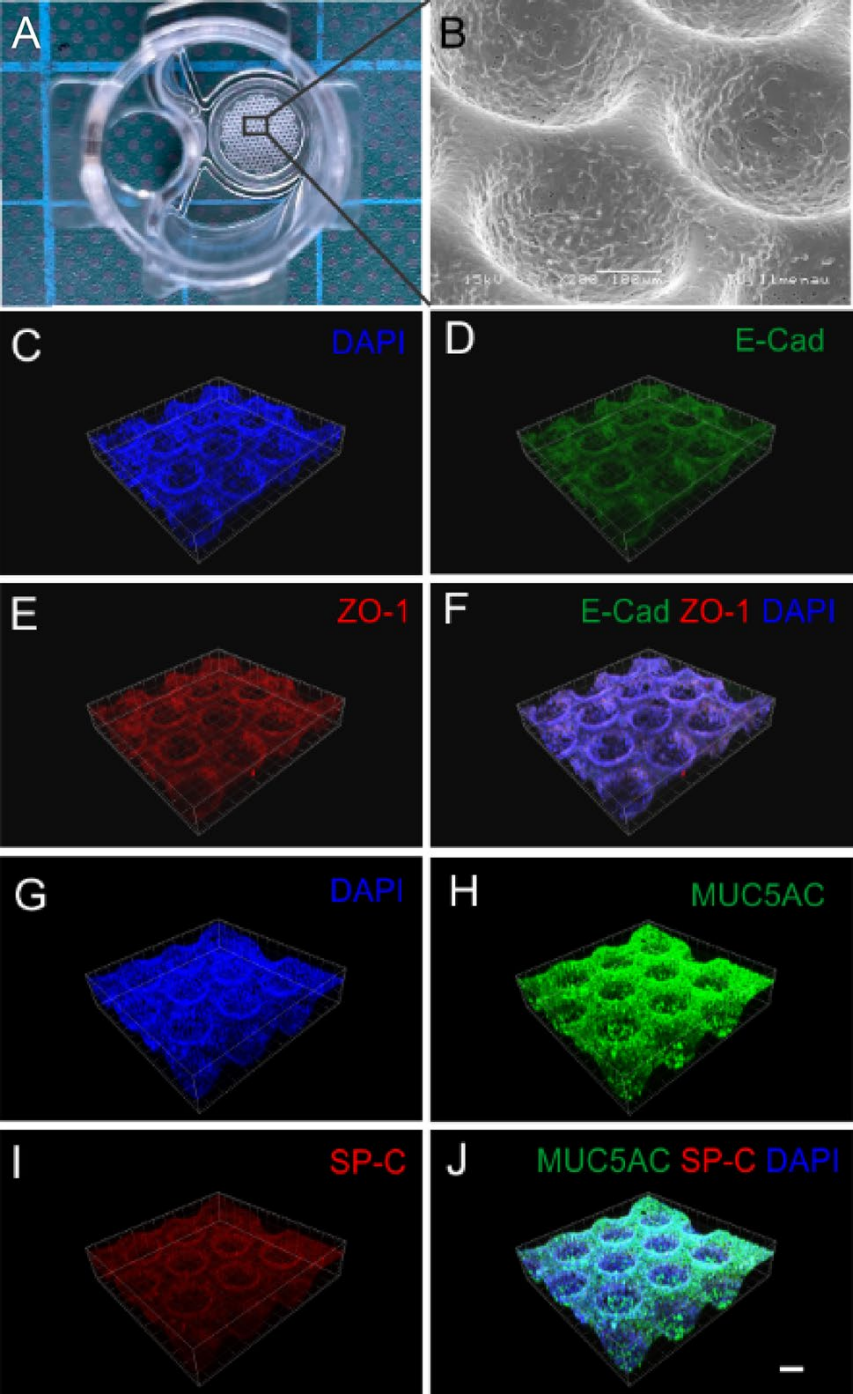
Characterization of a*ir–liquid-interface* (ALI)-cultured A549 cells in MatriGrids^®^: **A** semi active system with integrated MatriGrid^®^; **B** Scanning electron microscopy image of ALI-cultured 549 cells grown for 3 days. **C**–**F** Labeling of adherens junctions (E-cadherin) (**D**) and tight junctions (ZO-1) (**E**) of ALI cultures of A549 cells in MGs. **G**–**J** Production of mucus MUCIN 5AC (**H**) and surfactant protein-C (**I**) of ALI-cultured A549 cells grown in MGs for 3 days. Bar represents 100 µm

A549 cells were precultured under ALI condition for 3 days in MatriGrids^®^ (Fig. 6) for mimicking alveolar morphology. We have found that when using MALIES, MatriGrids^®^ are superior to standard transwell inserts because the cells do not dry out during the aerosol exposure. The MALIES creates a directed airflow into the well to place the nanoparticles precisely on the lung cells. A disadvantage of this type of exposure, however, is that the medium is easily displaced leading to impairment of cellular viability. In contrast, the cells in the MatriGrid^®^ cavities are soaked with medium during exposure and thus drying out of the cells is prevented. *Air–liquid interface* (ALI) cultures of A549 cells in MatriGrids^®^ (Fig. 6A) were investigated in terms of the formation of an epithelial barrier by scanning electron microscopy (SEM) (Fig. 6B) and the detection of adherens and tight junctions by zonula occludens (ZO-1)/E-cadherin staining (Fig. 6C–F). Unfortunately, parallel-performed transepithelial electrical resistence (TEER) measurements of cell layers were not analyzable due to the interference of the cavity morphology of the MatriGrids^®^ with the used measurement device. Additionally, MUC5AC- and surfactant protein C (SP-C)-expression of A549 type II pneumocytes was investigated by immunofluorescence staining to monitor mucus and surfactant production (Fig. 6G–J). Both SEM imaging and labeling of the junctional complexes revealed a developing epithelial barrier under ALI culturing of the cells in MatriGrids^®^. Furthermore, a strong production of mucus by the cell layers cultivated under ALI condition suggests adequate protection against nanomaterials. Additional surfactant protein production shows the ability to reduce the surface tension at the *air–liquid interface* and prevent collapse of alveolar cell layer.

To exclude possible effects of MatriGrid^®^-morphology on cellular properties and drug sensitivity compared with the common transwell-culturing method for lung cells, A549 cells were investigated under both cultivation conditions (MatriGrid^®^ and planar foil) (Supplement Fig. S1A). After precultivation under ALI condition on MG or planar foil for 3 days, cells were evaluated for their sensitivity against a test substance (CuSO_4_-solution) under submerged conditions (Fig. S1B), mucus and surfactant production (Fig. S1C) as well as their ability to form a barrier (Fig. S1D). We found no differences in the investigated cellular parameters as well as in the sensitivity against the test substance CuSO_4_ (see Fig. S1). Therefore, it can be assumed that the MatriGrid^®^ morphology plays a rather minor role in toxicity measurements with MALIES and can be used instead of transwell inserts. Aerosol exposure with CuSO_4_ of foil-cultured A549 cells using MALIES was not possible as the cells dried out during the 1 h exposure.

### Effects of BaSO_4_ and TiO_2_ nanoparticles on A549 cells in MatriGrids^®^

**Fig. 7:**
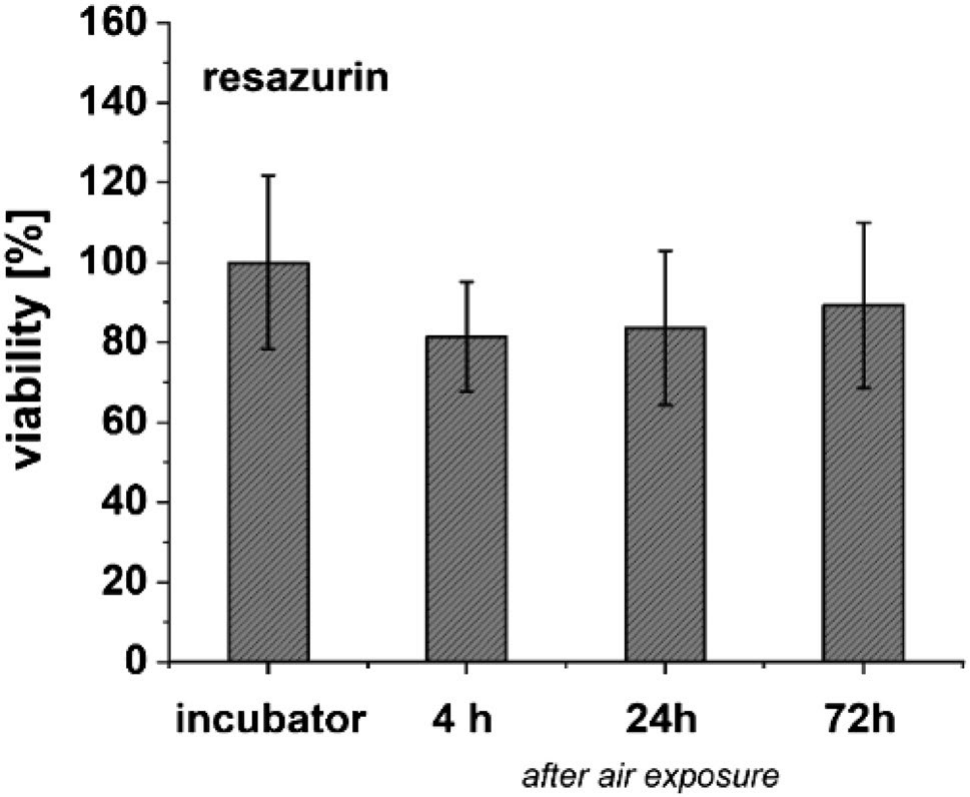
Effects of clean air exposure. Cell viability (metabolic activity) of ALI cultured A549 in MatriGrids^®^ cells after exposure with clean air for 1 h in the MALIES exposure system compared to the incubator control, which was cultured in parallel: Resazurin assay was performed 4 h, 24 h and 72 h after exposure with clean air. Shown are the mean values and standard deviations of n = 6 (4 h: n = 3) experiments

Before BaSO_4_- and TiO_2_-nanoparticle exposure, the effect of clean air exposure on ALI-cultured A549 cell viability (metabolic activity) was investigated with resazurin reduction 4 h, 24 h and 72 h after 1 h-exposure. Resazurin reduction of lung cells did not change markedly by air exposure compared to the incubator control (Fig. 7).

The exposure conditions for the experiments with BaSO_4_- and TiO_2_-NP (see Table 4) were above and below the maximum workplace concentration (for BaSO_4_-NP: 1.35 mg/m^3^ and for TiO_2_-NP: 1.27 mg/m^3^) (Forschungsgemeinschaft 2022). Due to the system specifications of MALIES (detection range of the SMPS, clogging of the aerosol generator) no higher nanoparticle yields could be achieved.

**Fig. 8:**
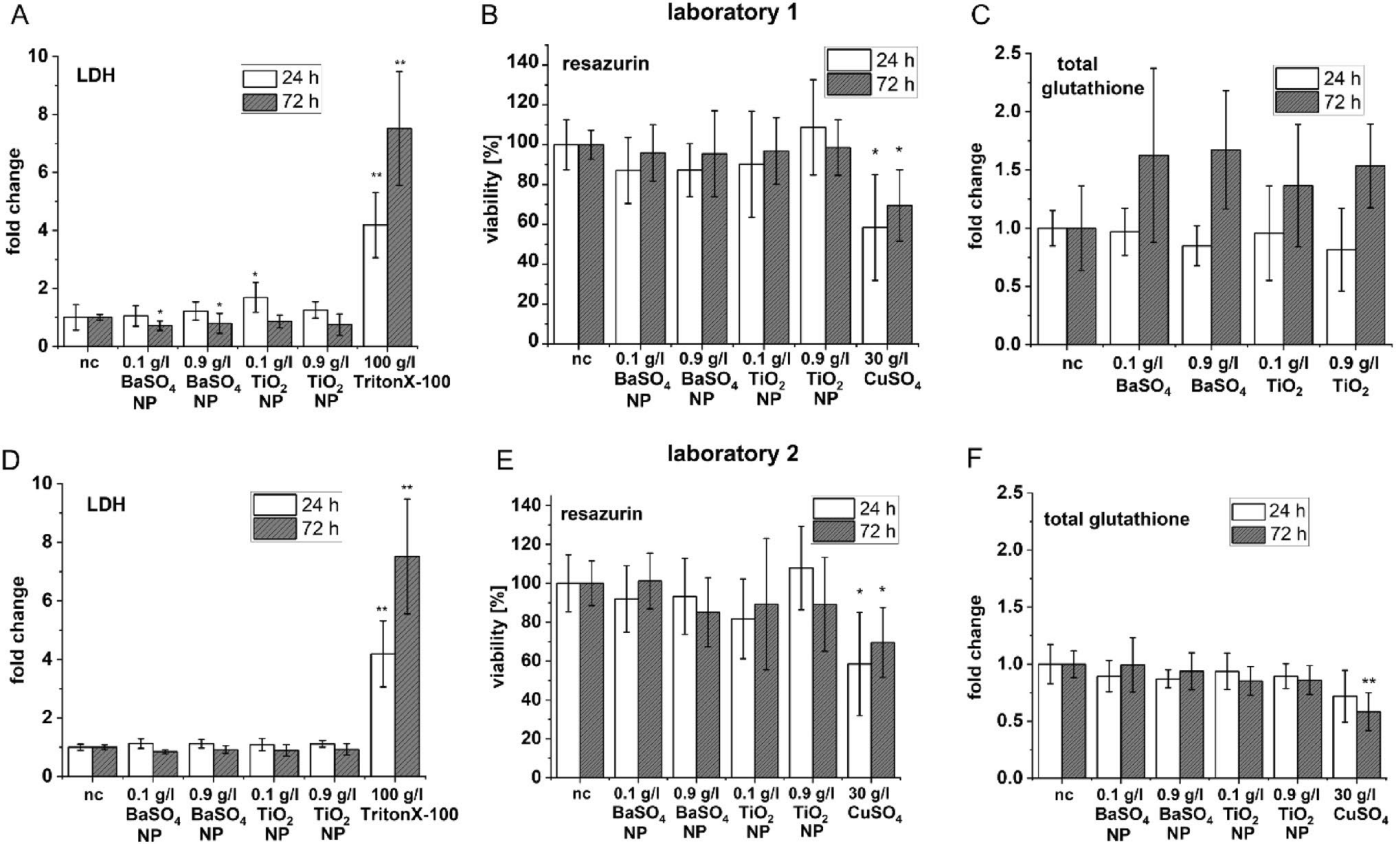
Aerosol exposure experiments with MALIES: **A**–**F** Comparative experiments with the MALIES device in two different laboratories. Low (0.1 g/l) and high (0.9 g/l) concentrations of BaSO_4_-NP and TiO_2_-NPs were exposed for 1 h to ALI-precultured A549 cells with the MALIES device and after 24 h and 72 h postincubation, LDH levels (**A**, **D**), resazurin reduction (**B**, **E**) and total glutathione levels (**C**, **F**) was determined in two independent laboratories. CuSO_4_ aerosol in a concentration of 30 g/l served as a positive control in resazurin and total glutathione determination with HPLC. Cells were treated in the same way as for NP exposure experiments. In LDH assay a 100% LDH release control (10% Triton × 100) was included. Shown are the mean values and the standard deviations of *n* = 3 experiments. *p ≤ 0.05; **p ≤ 0.01. nc = negative control with air exposure

A549 cells were exposed to nanoparticles with the MALIES exposure unit in MatriGrids^®^ for a duration of 1 h. Negative control A549 cells were exposed to air for the same duration. Analysis of the cytotoxicity of BaSO_4_- and TiO_2_-NP-aerosols at low (0.1 g/l) and high (0.9 g/l) suspension concentrations by the LDH assay revealed minimal increases in LDH in the culture supernatant 24 h after NP exposure at both laboratories (Fig. 8a, d), except for a significant 1.7-fold LDH release under 0.1 g/l TiO_2_-NP measured at laboratory 1 (Fig. 8a). In contrast, a positive control which induced a 100% LDH release (generated by Triton X100 treatment) showed a 4.2-fold increase in LDH in the culture supernatant. Prolonged cultivation of A549 cells until 72 h after NP exposure resulted in a decrease of LDH activity in the culture supernatant under both NP aerosols in both laboratories (for BaSO_4_-NP in laboratory 1 significant), suggesting a recovery of the cells (Fig. 8a, d), whereas 100% LDH release of A549 cells with Triton X-100 resulted in a 7.5 fold increase. In general, only a very slight and reversible damage of A549 cells by both NP aerosols can thus be assumed. Resazurin as a second cytotoxicity marker showed a similar course with minimal decreases in metabolic activity (between 9 and 18%) at NP suspension concentrations of 0.1 g/l and 0.9 g/l BaSO_4_ as well as 0.1 g/l TiO_2_ 24 h after exposure (Fig. 8b, e). A longer cultivation up to 72 h resulted in a recovery of the impaired metabolic activity. A simultaneous generated positive control in a concentration of 30 g/l CuSO_4_ showed a well measurable reduction of metabolic activity of 42% 24 h and 30% 72 h post exposure in the MALIES. The total glutathione content of A549 cells after NP-exposure was measured at laboratory 1 using a spectrophotometric method due to the lack of availability of an HPLC apparatus. In contrast to the results of laboratory 2, which could not detect any considerable differences in total glutathione by HPLC, 72 h after nanoparticle exposure at laboratory 1 slight increases in total glutathione values compared to the glutathione levels of the air-exposed control were detected, suggesting a possible adaptive cellular response to oxidative stress (Fig. 8c). In addition, very slight decreases in total glutathione levels 24 h after exposure to 0.9 g/l BaSO_4_-NP and TiO_2_-NP were also detected at laboratory 1 and for BaSO_4_-NP at laboratory 2, suggesting an initial reduction of total glutathione under these conditions. In parallel, CuSO_4_ aerosol in a concentration of 30 g/l induced a non-reversible significant reduction in total glutathione 72 h post exposure measured by HPLC. Unfortunately, parallel measurements on the formation of reactive oxygen species (ROS) worked poorly due to the intrinsic fluorescence of the MatriGrids^®^, so that these data are not presented here. In addition, releasing the cells from the MatriGrids^®^ did not improve the results due to the instability and rapid decay of the reactive oxygen species.

## Discussion

We present a novel aerosol exposure system (MALIES), which can be used to study the effect of nanoparticle aerosols on 3D-cultured lung cells. MALIES is a modular device whose individual components for aerosol generation, conditioning, distribution and exposure are easy to acquire and assemble. The components of MALIES can be replaced according to the user's requirements. Depending on the test substance to be aerosolized, rotary scrapers or venturi generators for e.g. carbon nanotubes can be used instead of the Pariboy for nanoparticle suspensions (Polk et al. 2016). The 4-channel exposure unit can be used with standard micro titer plates containing semi-active systems with alveolus-like scaffolds made from polycarbonate, the MatriGrid^®^. The special MatriGrid^®^ morphology with its container like cavities largely protects the inner cell layer from drying out during ALI culture and in particular during aerosol exposure with MALIES. Here, supplementary investigation revealed that in combination with MALIES MatriGrids^®^ are superior to transwell inserts. Furthermore, sensitivity to CuSO_4_ solution, barrier formation and secretion of mucus and surfactant of MatriGrid^®^ cultured lung cells did not differ from that of cells cultured on planar foil indicating that cavity morphology has no influence on cellular properties. Different types of lung cells, together with other lung resident cells can be (co)cultured on this scaffold under *air–liquid interface* conditions and used for nanoparticle aerosol exposure; e.g. cocultures of pneumocytes with endothelial cells (EC). ECs can be placed on the outside of the MatriGrid^®^ and pneumocytes on the inner side forming the alveolar capillary interface. In addition, lung-resident alveolar macrophages, which are responsible for clearance of pathogens, nanoparticles, surfactant and cell debris (Hu and Christman 2019), can be seeded in the microcavities near the alveolar epithelial cells. Pores in the polycarbonate MatriGrid^®^ scaffolds enable passage of medium from the lower to the upper compartment and interactions of any cocultures used through paracrine growth factors and signaling molecules. Compared to other aerosol exposure devices, which work with nebulization and gravitational settling (Aufderheide and Mohr 2000; Barosova et al. 2020; Braakhuis et al. 2020; Gervelas et al. 2007; Hufnagel et al. 2020; Niwa et al. 2007) or electrostatic precipitation for particle deposition (de Bruijne et al. 2009; Frijns et al. 2017; Mülhopt et al. 2016), MALIES uses individual nozzles over every MatriGrid^®^ ensuring even distribution of the nanoparticle aerosol. Moreover, the system includes a diffusion dryer, which removes excess liquid in the aerosol, which normally interferes with the precise characterization of the aerosols. Furthermore, dehumidifaction of suspension-based aerosol better imitates ambient air contaminated with nanoparticles, as nanoparticles typically appear as dry, aerosolized nanoparticles (Upadhyay and Palmberg 2018).

Our simulation of particle distribution in the applied semi-active systems revealed only a small amount of vortex formation at the nozzle above the MatriGrid^®^. Furthermore, based on the measurement with fluorescent micro particles, we can show, that the exposure unit, we developed, distributes the aerosol particles evenly over the four channels. This serves as an essential prerequisite for reliable application in cell exposure experiments. MALIES was successfully tested for its ability to induce a dose-dependent toxicity in A549 cells upon aerosol exposure with increasing concentrations of CuSO_4_. In addition to a well detectable increasing deposition of copper on the MatriGrids^®^ after aerosol exposure, a dose-dependent reduction in the viability of the A549 cells was found in the resazurin assay.

SMPS particle measurements were used to determine the mass flow rate for the nanoparticles. The calculations showed that a high proportion (at least 75%) of the generated particles are deposited in the exposure system on their way to the nozzle above the MatriGrid^®^. Final nanoparticle aerosol concentrations in the air above the MatriGrid^®^ were 0.29 mg/m^3^ BaSO_4_-NP, 0.84 mg/m^3^ TiO_2_-NP in the low (0.1 g/l) and 3.15 mg/m^3^ and 10.90 mg/m^3^ in the high exposure group (0.9 g/l).

Additionally performed TEM measurements revealed that nanoparticles were deposited on TEM carriers after one-hour aerosol exposure, which makes an interaction with the A549 lung cells on MatriGrids^®^ likely. Depending on the nanoparticle concentration used in the aerosol formation, a corresponding lower or higher number of nanoparticles were detected on the TEM carriers (0.1 g/l NP-suspension: 0.3 µg BaSO_4_-NPs and 0.6 µg TiO_2_-NPs; 0.9 g/l NP-suspension: 8.1 µg BaSO_4_ and 22.2 µg TiO_2_). It was found that more TiO_2_-NP than BaSO_4_-NP were finally deposited on the carriers (twofold for the exposed concentration of 0.1 g/l and 2.7 fold for 0.9 g/l nanoparticles compared to BaSO_4_-NP).

This could be due to increased aggregation of BaSO_4_-NP during the aerosol exposure procedure, and possibly increased deposition of particles in tubing and aerosol paths of the MALIES leading to increased losses of BaSO_4_ nanoparticles in the system. Moreover, as the mean aerodynamic diameter of the TiO_2_ particles was larger, a higher mass was transported onto the grids.

The deposition efficiency of MALIES was calculated via TEM data and was in the range of 2.6–8.9% depending on the inlet particle concentrations. The maximum deposition efficiency was obtained for BaSO_4_-NP with 8.9%. Deposition efficiency of MALIES is higher than that of the CULTEX-type exposure systems [2% (Bitterle et al. 2006) and 0.05% (Elihn et al. 2013)] and comparable to that of the Vitrocell-type (7–22%) (Loret et al. 2016). Other aerosol exposure systems, which work with electrostatic deposition, achieve much higher deposition efficiencies of 35–47% and 75–95%, respectively (de Bruijne et al. 2009; Frijns et al. 2017). However, it is not clear how these exposure systems change the original properties of the particles and thus their effect on cells. In addition, the deposition rate is also influenced by the type of nanomaterials that are used.

Final deposited nanoparticle doses generated by MALIES ranged between 0.9 and 74.0 µg/cm^2^ for the different nanoparticles (BaSO_4_-NPs and TiO_2_-NPs). The deposited masses for TiO_2_-NP obtained with MALIES are higher than those compared to Loret et al. (2016), where deposited masses of 0.1–3 µg/cm^2^ were achieved and deposited masses published in Hufnagel et al. (2020) (up to 25.8 µg/cm^2^). Both were using Vitrocell exposure systems. BaSO_4_-NP have not yet been used in aerosol exposure systems and no comparative data are available.

NP sizes measured by SMPS were stable in the nm range according to the recommendations of the European Commission (2022). BaSO_4_-NPs of the high exposure group (0.9 g/l) differed in the two laboratories where a higher proportion of NPs with a size of 100 nm in laboratory 2 were found.

Exposure of A549 cells with clean air to investigate the impact of the exposure system on cell viability showed a slight impairment of the resazurin reduction of A549 cells 4 h after exposition, which regenerated with increasing incubation time. Decrease of metabolic activity of cells can be explained by the influence of the air flow on the underlaying cell layer (non-physiological pressure and slight displacement of culture medium) and was also oberved in other aerosol exposure systems (Kim et al. 2013; Lenz et al. 2009; Savi et al. 2008).

Nanoparticle toxicity data obtained in both laboratories demonstrated the reliability of the MALIES setup and comparable results. BaSO_4_-NP aerosols in deposited doses of up to 26.6 µg/cm^2^ and TiO_2_-NP aerosols in deposited doses of up to 74 µg/cm^2^ did not induce notable cytotoxicity in A549 cells measured by LDH activity and resazurin reduction 24 h after exposure. The observed minimal changes were reversible 72 h after exposure. In contrast, CuSO_4_ aerosol in a deposited dose of 37.7 µg/cm^2^ was able to produce a pronounced cytotoxicity in A549 cells 24 h post exposure.

Measurement of total glutathione content showed the same result with no significant changes except for laboratory 1. A possible reason for the increase of total glutathione levels in laboratory 1 might be additional stress caused by the slightly more pronounced changes in pH values in these experiments, which are due to the different buffer systems used. In laboratory 1 HEPES in the medium is used to keep the pH value, while in laboratory 2 CO_2_ is continuously supplied in the aerosol. The positive control CuSO_4_ caused a non-reversible decrease in the amount of total glutathione in HPLC 72 h post exposure, which may be due to increased degradation/depletion, efflux or decreased new synthesis of the antioxidant tripeptide in response to oxidative stress by CuSO_4_.

Our results with the nanoparticles are consistent with the in vitro study of Kroll et al. (2011), who observed a very slight effect of 10 µg/cm^2^ BaSO_4_-NP on A549 cells with the MTT assay. Here a significant reduction of metabolic activity could only be detected in NIH-3T3 fibroblasts. If we use our calculated air concentrations of BaSO_4_ (3.15 mg/m^3^) during aerosol exposure, we are clearly below the concentrations reported in the literature at which an overload situation in rats can be expected (50 mg/m^3^) (Konduru et al. 2014; Molina et al. 2019) or inflammatory responses are induced in rats (Konduru et al. 2014). Thus, nanoparticle concentration generated by MALIES were too low to compare our in vitro data with the published in vivo data obtained in rats. Furthermore, we used short-term exposure conditions compared to the in vivo studies which run over weeks (Konduru et al. 2014; Molina et al. 2019). However, our data show that even minor changes of cell viability induced by BaSO_4_-NP can be demonstrated in A549 cell culture model using MALIES.

Exposure to TiO_2_-NP aerosol resulting in deposited masses of 2.1 and 74.0 µg/cm^2^ was also not or only slightly cytotoxic to A549 cells with a significant 1.6 fold increase of LDH in response to TiO_2_-NP in the low exposure group in the laboratory 1. (Hufnagel et al. 2020) showed that up to deposited doses of 25.8 µg/cm^2^ TiO_2_-NP, there were no effects on the expression of genes related to metal homeostasis, oxidative stress response, apoptosis and DNA-damage in A549 cells. Similarly, (Loret et al. 2016) found in their study no effect of TiO_2_-NP aerosol using doses from 0.1 to 3 µg/cm^2^ on cellular functionality and integrity, but induction of proinflammatory markers like IL-6, IL-8 and TNFalpha in A549/THP-1 cocultures. Our TiO_2_-NP dose of the high exposure group (0.9 g/l) with a deposited mass of 74.0 µg/cm^2^ exceeds these NP doses and is more comparable to the study by Rach et al. (2014). They observed a significant decrease in cellular viability (up to 70%) at a dose of 25 µg/cm^2^ Aeroxide^®^ TiO_2_-P25 nanoparticles per 15 min using a CULTEX radial flow system device in a bronchial epithelial cell line. The difference in cellular outcome could be due to the type of nanomaterial used (shape, size) and cell type. Considering the air-aerosol concentration of TiO_2_-NP of the high exposure group (10.90 mg/m^3^) generated by MALIES, it corresponds to the aerosol overload situation determined in vivo in long-term experiments with rats, which is 10 mg/m^3^ (Landsiedel et al. 2014a). However, no direct comparison is possible since the duration of exposure of 5 days differed significantly from our one hour exposure time.

## Conclusion

In the present study, the short-term cytotoxicity of BaSO_4_-NP and TiO_2_-NP aerosol on MatriGrid^®^-cultured alveolar epithelial cells at the *air–liquid interface* was investigated using the MALIES setup. The deposition efficiency of MALIES ranged from 3.2 to 8.9%, and the dose of deposited nanoparticles from 0.9 to 74.0 µg/cm^2^. The cytotoxicity data obtained are comparable to previously published results by other researchers who found no significant effects of these nanoparticle concentrations on cell viability and no persistent toxic effects in rats. To our knowledge, this is the first study investigating the effects of BaSO_4_-NP aerosol on alveolar epithelial cells using an aerosol exposure system. We found only minor and reversible effects of short-term treatment with BaSO_4_-NP aerosol on metabolic activity, cell integrity and oxidative stress response. However, the aerosol concentrations produced by MALIES were far below the overload concentration for BaSO_4_-NPs.

By dehumidifying the NP aerosol, MALIES allows accurate determination of the concentration of nanoparticles in the aerosol air, while also preserving the physicochemical properties of the particles. A drawback of this approach is the generation of a dry particle stream, which in turn can have a desiccating effect on the exposed cells. However, this drying stress can be alleviated by cultivating the lung cells in microcavities of the MatriGrid^®^, whereby the cells are humidified during the exposure scenario.

In future, further precautions should be taken to minimize cell drying during the exposure process, such as the continuous supply of medium at the *air–liquid interface* in the MALIES setup.

To better compare the generated data with in vivo toxicity studies in animals, the nanoparticle deposition rate and thus the nanoparticle dose on the cells in the MALIES setup must be increased. Furthermore, it is essential to apply nanoparticles chronically and repeatedly to better mimic environmental exposure scenarios. An essential prerequisite for a better predictability of nanoparticle toxicity and safe application is the use of primary cells, cocultures or miniorgan cultures (MOCs)/lung explants in aerosol exposure experiments.

### Supplementary Information

Below is the link to the electronic supplementary material.

Supplementary file 1 (DOCX 1018 KB)

## Data Availability

The data can be obtained from the corresponding author on request.
