# Supplementary material for: Modular air–liquid interface aerosol exposure system (MALIES) to study toxicity of nanoparticle aerosols in 3D-cultured A549 cells in vitro
Source: Arch Toxicol. 2024 Feb 10;98(4):1061–80. doi: 10.1007/s00204-023-03673-3 (PMC10944414; doi:10.1007/s00204-023-03673-3)
Supplement: Supplementary file 1 — (DOCX 1018 KB) [file 204_2023_3673_MOESM1_ESM.docx]

**Supplementary Information (SI)**

Materials and Methods

Air-liquidinterface (ALI) culture of A549 cells in semi-active systems containing porous polycarbonate foil (PC-foil) or MatriGrid^®^

Semi-active systems containing PC foil and MatriGrids^®^ were sterilized like described in the main text (material and methods) and then transferred to 24-well plates. 500 µl medium was added to the basal side of the PC foil or MatriGrid^®^. 25 µl of a cell suspension containing 1.35×10^5^ A549 cells per cm^2^ were seeded onto the apical side of the PC foil or scaffold. Cells adhered to the different polycarbonate surfaces for one hour at 37 °C and 5 % CO_2_ in the cell incubator. Thereafter, 500 µl medium was added to the apical side of the PC foil or MatriGrid^®^. Inserts with A549 cells were incubated for 24 h under submerged conditions (500 µl medium each on the apical and basal side of the MatriGrid^®^ in the insert), followed by incubation at the *air-liquid interface* (ALI, 500 µl medium on the basal side) for three days.

Determination of CuSO_4_-sensitivity of A549 cells cultured on planar PC-foil and MatriGrid®

ALI-precultured A549 cells on porous foils and MatriGrids® were incubated in serum-free DMEM medium supplemented with 1 % sodium pyruvate, 2 % glutamine and 100 U/ml penicillin/ 100 µg/ml streptomycin (Pen/Strep) containing CuSO_4_ (Carl Roth; Karlsruhe; Germany) in concentrations of 0, 0.02, 0.04, 0.08, 0.12, 0.16, 0.20 and 0.24 g/l) for 24 hours. Subsequently A549 cells were washed with fresh medium without CuSO_4_ and the resazurin assay for determination of the viability was performed. Therefore, cells were incubated with 10 % Alamar Blue solution for 1 h at 37 °C. The concentration of resorufin generated by A549 cells was determined by fluorescence spectrometry (ex: 530 nm; em: 590 nm) in a plate reader (Spectramax, Molecular Devices, San Jose, USA). Relative viability was calculated by normalization of measured values from nanoparticle exposed samples to the value of air-exposed control which was set to 100 %.

MUC5-AC/SP-C and ZO-1/E-cadherin immunofluorescence

Immunofluorescence staining of A549 cells cultured on PC foil or MatriGrid were performed like described in the material and methods chapter of the main text. The same antibody-combinations (MUC5AC/SP-C and E-Cadherin/ZO-1) and staining conditions were used. Images were taken with an OLYMPUS laser scanning microscope FV1000 (Olympus, Germany).

Results


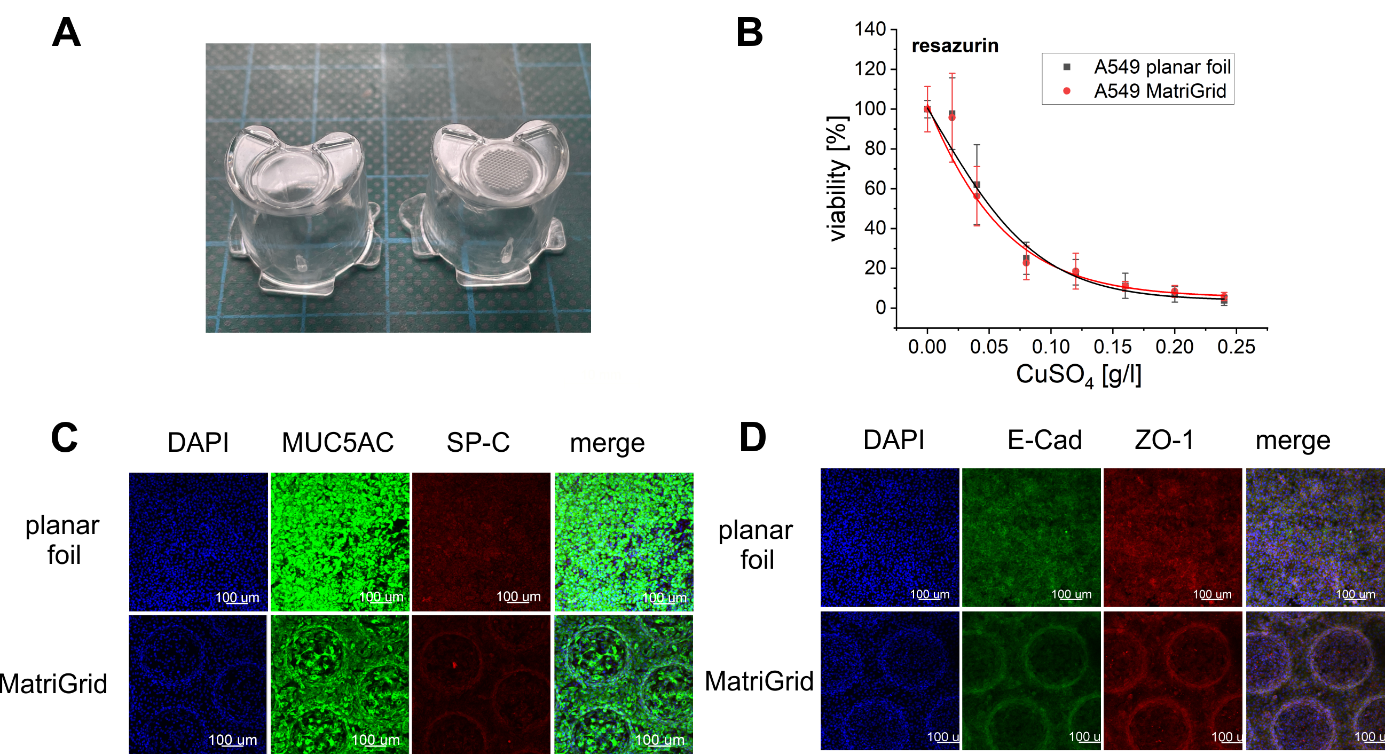


**Fig. S1: Comparison of A549 cells cultured on porous foils or in MatriGrids^®^: (A)** Porous polycarbonate (PC) foil (left) and MatriGrid^®^ (right) formed from PC foil embedded in semi active systems **(B)** For 72 h in ALI precultured A549 cells were treated with increasing concentrations of CuSO_4_-solution (0, 0.02, 0.04, 0.08, 0.12, 0.16, 0.20 and 0.24 g/l) for 24 h. Viability was determined via resazurin reduction and normalized to the untreated control value which was set to 100 %. Shown are the mean values +/- SD (n = 3). **(C)** Production of mucin (MUC5AC) and surfactant protein (SP-C) by A549 cells cultured in ALI for 72 h on PC foil or MG. **(D)** Labeling of adherens junctions (E-cadherin) and tight junctions (ZO-1) of ALI cultures of A549 cells on PC foil and in MGs. n = 3; Bar represents 100 µm.
